# Integration of Directional Antennas in an RSS Fingerprinting-Based Indoor Localization System

**DOI:** 10.3390/s16010004

**Published:** 2015-12-23

**Authors:** Raúl Guzmán-Quirós, Alejandro Martínez-Sala, José Luis Gómez-Tornero, Joan García-Haro

**Affiliations:** Department of Information and Communication Technologies, Universidad Politécnica de Cartagena, Cartagena 30202, Spain; alejandros.martinez@upct.es (A.M.-S.); josel.gomez@upct.es (J.L.G.-T.); joang.haro@upct.es (J.G.-H.)

**Keywords:** indoor localization system, received signal strength, fingerprinting technique, artificial neural networks, directional antennas

## Abstract

In this paper, the integration of directional antennas in a room-level received signal strength (RSS) fingerprinting-based indoor localization system (ILS) is studied. The sensor reader (SR), which is in charge of capturing the RSS to infer the tag position, can be attached to an omnidirectional or directional antenna. Unlike commonly-employed omnidirectional antennas, directional antennas can receive a stronger signal from the direction in which they are pointed, resulting in a different RSS distributions in space and, hence, more distinguishable fingerprints. A simulation tool and a system management software have been also developed to control the system and assist the initial antenna deployment, reducing time-consuming costs. A prototype was mounted in a real scenario, with a number of SRs with omnidirectional and directional antennas properly positioned. Different antenna configurations have been studied, evidencing a promising capability of directional antennas to enhance the performance of RSS fingerprinting-based ILS, reducing the number of required SRs and also increasing the localization success.

## 1. Introduction

Many context-aware applications require services dependent on the user/tag location [1]. To name a few, guide navigation in museums and malls or asset tracking for logistical purposes in hospitals, airports or general industrial environments are some of the most promising services currently demanded. Most of these applications usually require indoor location, where global navigation satellite systems (GNSS) cannot guarantee location service due to the obstruction of the satellites’ signals [2]. For this reason, alternative indoor localization systems (ILS) employing different technologies are increasingly drawing more attention, [3]. Most of them are differentiated by the type of metrics employed to infer the position, such as time of arrival (TOA), time difference of arrival (TDOA), angle of arrival (AOA) or received signal strength (RSS). Systems that incorporate TOA, TDOA or AOA measurements usually achieve high location accuracy with errors below 1 m, but at the expense of more complex and expensive devices than RSS solutions due to the need for complex synchronization schemes and higher bandwidth requirements [4,5,6,7,8].

Currently, solutions based on RSS are quite attractive because of their easy integration in wireless networks and extremely low cost, since RSS measurement is usually reported by most of the current standards [9]. Basically, in the literature, three different techniques can be found to estimate the position from the RSS. (1) Proximity algorithms [10] provide symbolic relative location information. In these cases, the tag position is associated with the known location of the sensor that measures the strongest RSS value. They usually rely on a dense grid of sensors; (2) Distance-based methods [9,11,12,13,14] require estimating the distance between the tag and the sensors from the RSS, obtaining the tag location by multilateration algorithms [9]. However, a good radio propagation model of the indoor electromagnetic channel is required [12,13,14,15], so the greater precision of the model, the better location accuracy achieved. These models are not trivial and usually require a custom-made survey of the indoor environment under consideration to obtain the model parameters. Finally; (3) the third group of systems encompasses those ones based on the fingerprinting-based technique (also called scene analysis) [16,17,18,19]. Fingerprinting has become one of the most popular and extended techniques applied to RSS-based indoor positioning systems. This technique mandatorily requires a site survey of the particular scenario of interest in order to gather a set of fingerprints composed of RSS vectors associated with the possible space locations where the tag can be positioned. Thus, a fingerprint database is created, which is generally called a radio map.

Several approaches based on different architectures and positioning algorithms [4] have been proposed to estimate the tag position from the radio map. Among others, the most outstanding algorithms are maximum likelihood (ML) [20,21,22], K-nearest neighbors (KNN), weighted KNN (WKNN) [11] and diverse learning machines, such as support vector machines (SVMs) [23,24], radial basis functions (RBF) or artificial neural networks (ANN) [25,26,27,28,29]. ML, KNN or WKNN consist of comparing gathered RSS vectors with the RSS fingerprints stored in the radio map. Thereby, the most likely tag position is figured out. On the other hand, machine learning algorithms need an intermediate training phase after the site survey and before the operating stage. The machines must be previously trained so they can learn the mapping function between RSS signal space vectors and spatial locations in the radio map. After the training process, these learning machines will act as classifiers, providing the most probable location according to the knowledge acquired.

Independent of the inferring technique, the unpredictable temporal fluctuation of the RSS inside indoor buildings is one of the main issues that any RSS-based ILS encounters and which jeopardizes their robustness and maximum accuracy [16,17]. These irregularities of the electromagnetic channel are produced by uncontrolled factors, such as interfering electric/electronic devices, people moving, open/closed doors, temperature/humidity variation as a function of the daytime/climatology and other dynamic phenomena [29,30]. Due to these phenomena, in-building location estimation from RSS is a complex problem that is difficult to be engineered using classical mathematical methods. For this reason, recent works have tried to incorporate improvements to filter the RSS noise sources and finely tune the tag position by combining different techniques [31,32,33,34,35,36,37,38], introducing additional sensor data (e.g., digital compass or pedometers [29,31], accelerometers [36,37,38], *etc.*), employing Kalman or particle swarm filters [32,33,34,35] or improving the radio map quality [18,19].

Nonetheless, it is interesting to note that RSS fingerprinting-based indoor localization techniques exploit the RSS variability, but from the spatial perspective, *i.e.*, the greater the RSS changes along space, the more robust and the easier is the fingerprinting-matching task, as more dissimilar fingerprints arise at each possible tag location; hence, the higher the accuracy that can be achieved [16]. Indeed, this spatial variance can be interpreted as a negative effect, which increases the overall RSSI variance and degrades the localization performance, but this interpretation is not strictly right. The proper indoor channel complexity (mainly given by phenomena, such as non-line of sight (NLOS) multi-path effects because of reflections/refractions due to the building geometry, wall/floors, building material losses, *etc.*) can be an advantageous property for these systems, as it increments this spatial variation in the RSS distribution compared to outdoors, where RSS decay is mainly predicted by the distance losses with respect to the source.

In this paper, we study the employment of omnidirectional and directional antennas to improve the accuracy of an RSS fingerprinting-based ILS for room-level positioning. The hypothesis behind it is: the variance of the RSSI (in terms of space) could be increased by integrating suitably-positioned and -oriented directional antennas (more directive antennas introduce higher sensitivity/variance with space). Thus, increased spatial variability should create more dissimilarity between different locations (fingerprints); the radio map quality can be enhanced, as fingerprints will be more robust to noise (generated by the aforementioned dynamic phenomena) and, hence, more easily associated with the location. The rest of the paper is organized as follows. Related work is provided in Section 2. System operation concepts and architecture are introduced in Section 3. Section 4 describes the system deployment cycle, focusing on the description of the sensor network planning and calibration phases. Finally, Section 5 and Section 6 discuss experimental test results and conclusions, respectively.

## 2. Related Work

Several works have proposed learning machines as good candidates to estimate the tag position from RSS measurements. In [24], a kind of artificial neural network, called multilayer perceptron (MLP), was proposed to map for the first time the relation between coordinates (*X,Y*) of the tag position and the RSS gathered by an *ad hoc* WLAN. In [23,24,25,26,27], other types of learning machines, such as support vector machines (SVM), learning vector quantization (LVQ) and radial basis functions (RBF), were respectively studied for the same purpose, obtaining similar results. Up to date, most of these systems offer a coarse precision of 3–6 m for 90% of estimations [4], if no additional information/sensors are integrated [16]. This precision can be acceptable for many location-based services (LBS), which require room-/area-level localization [13,21,22,23], but not for exact point location. In fact, room-level accuracy has high interest given the number of highly practical applications that can be integrated with low cost techniques and quite extended commercial systems (smartphones, tablets, *etc.*). However, there is still a problem with service reliability, because of the RSS temporal fluctuations. At this point, it is good to note that the presented work is in the frame of the Spanish Project “ChAracterization, EvaLuation, Planning and IMprovement of Key Technologies for the Future Internet: Knowledge and Transfer” (CALM), Project Ref. TEC2010-21405-C02-02, which was intended to provide room-level localization of patients inside a hospital. In many particular applications as this one, meter/submeter indoor positioning is not necessary, and less accurate, much more inexpensive room-sized localization is adequate. This type of room-level solution can be found in many commercial products; see for instance [39,40,41,42,43].

Several previous works have proposed different approaches to provide room-level positioning so far, which seem to have obtained good results. In [13], a coarse room-level accuracy is given based on selecting the anchor node with the maximum associated RSS. Each anchor is associated with a room. However, problems arise when the tag is close to walls. This issue is improved in [21], where a path-restricted room-level localization system is proposed with additional intelligence, achieving an average success probability of 94%. Furthermore, high success is achieved in [22,23], where RSS-based probabilistic algorithms are applied to refine the room-level tag location based on detecting tag distance to passable boundary points (e.g., at entrances) [22] or calculating a transition probability between points [23], so they detect when the tag is likely moving between predefined areas with additional post-processing techniques. According to the experiments, around 95% of success is the average in [22] and 97% in [23], respectively, in controlled conditions.

However, all previous cited works assumed omnidirectional antennas for the sensor readers and tags, obviating the interesting properties of non-isotropic (directional) antennas. Unlike omnidirectional antennas, directional antennas may report better spatial discrimination, because of their higher directivity at some angular directions. Some works have proposed exploiting directional antenna properties in a different way [44,45,46,47,48,49,50,51,52]. In [44], a review of antennas applied for indoor localization systems can be found. An AOA-based technique was presented in [45], where the angle was estimated from the difference between RSS captured by two directional antennas. Furthermore, [46,47,48,49,50,51,52] present similar AOA-based techniques employing directional antennas for location estimation in wireless sensor networks (WSN), reducing to two the minimum number of anchor nodes (sensor readers (SRs)) to estimate the tag position by multilateration techniques. In particular, in [52], the tag position was estimated from TOA and AOA measurements, the last one being estimated from the RSS received by an array of directional antennas.

None of these works have analyzed the integration of directional antennas to enhance the performance of a room-based RSS fingerprinting-based ILS following the hypothesis laid down in this paper, *i.e.*, the increment of the spatial variability of the RSS distribution through intrinsic directional antennas’ spatial variability. As will be seen below, a classical room-level RSS-based positioning engine based on a simple MLP classifier will be employed to carry out the study. Omnidirectional and directional antennas have been integrated in order to analyze their effect on the system performance. In the next section, the architecture of the system is presented in detail.

## 3. Proposed Indoor Localization System

**Figure 1:**
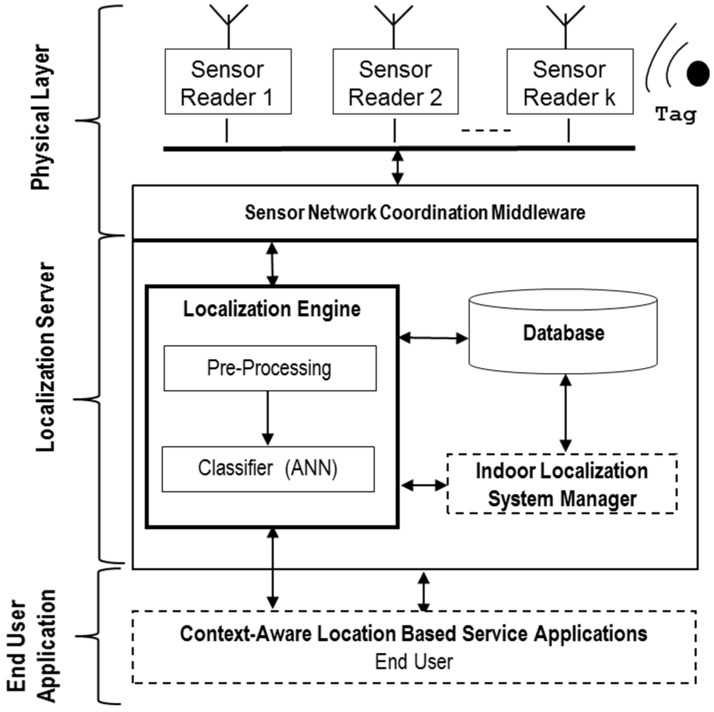
Block diagram of the proposed RSS-based indoor localization system (ILS).

The block diagram of the proposed system is basically composed of three blocks, as illustrated in Figure 1: (1) the physical layer, where the RSS is captured by the sensor reader (SR) network and managed by the sensor network coordination middleware (SNCM); (2) the localization server, in which the RSS data are processed in order to obtain the final position of the tag; and (3) the end user application block, which represents end users. Herein, context-aware applications demand the location information to be employed by LBSs.

The sensor network consists of a set of *K* sensor readers (SRs) placed at different fixed locations throughout the scenario. These devices are in charge of capturing the RSS from the beacons transmitted by the tag, which can be located in some of the *M* rooms or areas of interest (***R*** = {*R_1_,...,R_i_,...R_M_*}) into which the scenario was previously divided. Then, the tags whose position is desired will periodically send *N* beacons, and their RSS will be captured by the SRs. Each *k*-th SR creates an RSS vector with *N* components, corresponding to each transmitted beacon: *S* = {*S_1_,...,S_j_,...S_N_*}. The captured RSS vector is organized by the SNCM layer, guaranteeing data integrity and generating a final ***N*** × *K* RSS data matrix, which will be forwarded to the localization server (second block) to infer the tag position.

The localization server is mainly responsible for estimating the room ID (*R_i_*) where the tag is positioned. This localization engine constitutes the fundamental part of this block and consists of a two-layered architecture, which sequentially processes the RSS data matrix generated by the SNCM in the physical layer to estimate the most probable room where the tag might be located. The first layer corresponds to a pre-processing of the raw data and basically filters out outliers and averages the captured RSS data from each SR. After the pre-processing, the classifier layer, including the localization algorithm (an ANN, as will be seen), processes the averaged RSS and infers the room ID. Furthermore, a database is integrated with the server. This is employed to store all of the information related to the radio map fingerprints, system configuration and parameters related to the artificial neural network (ANN). The indoor localization system manager is the software tool supported by the server, implemented in C# and MATLAB. This program allows one to control the whole system, configuration, tag parameters, calibration process, ANN training, *etc.*, giving flexibility and agility to manage the system offline phase (deployment, radio map generation and system calibration) and the experimental tests easily, as will be shown in the next sections.

Finally, this information will be accessed by the context-aware services when demanded by the end users, in the third block. Therefore, the system can be seen as a platform to provide positioning information to support different context-aware applications (tracking, surveillance, assets control, *etc.*).

## 4. Deployment Cycle

**Figure 2:**
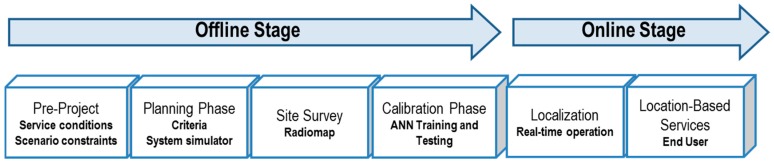
System deployment cycle.

In order to setup the RSS fingerprinting-based ILS, the deployment cycle depicted in Figure 2 has been considered. Basically, it can be divided into two stages, which are common for any kind of fingerprinting-based ILS, the offline and the online stages. The offline stage is dedicated to setup the system, *i.e.*, plan the antennas’ initial position, generate the radio map (site survey process [1]) and train the ANN. Otherwise, in the online stage, the system is operating in real time, estimating and providing the tag localization to the end user.

Regarding the offline stage, this is divided into four phases, namely: Pre-project, planning, setup and calibration phases. Firstly, the pre-project phase fulfils an analysis of the indoor scenario in detail, in order to define the rooms/areas of interest where the tag will be localized (imposed by the service conditions) and find the restrictions imposed by the infrastructure itself, such as the location of its power network sockets to feed the system or the potential electromagnetic interference sources, which could interfere with the ILS. Secondly, the planning phase is devoted to initially positioning the SR antennas according to the service conditions and restrictions. Thirdly, the site survey is the process by which the radio map is generated. In this step, a number of fingerprints are generated in order to calibrate the system in the fourth phase, where the ANN is trained and tested with the radio map, so that it can learn to map the RSS vectors with the location of the tag. The latter phases are neatly explained in the next subsections.

### 4.1. Site Survey and Calibration Phase

One of the drawbacks of any fingerprinting-based ILS is the offline stage. This stage requires time and manpower. In addition, this process must be repeated for every scenario where the system is set up or if the infrastructure suffers major modifications. For this reason, some authors are looking for research lines to deal with the reduction of the radio map generation efforts [18] or trying to develop systems that omit this step totally or partially as much as possible [53].

#### 4.1.1. Radiomap Generation

The site survey must be performed in order to generate the radio map. This map allows one to characterize the spatial RSS distribution inside the indoor environment, collecting the so-called fingerprints, which in this case are composed of an RSS data matrix with *N* × *K* features (*N* transmitted beacons captured by *K* SRs) and the associated location (room ID) from where the tag transmitted the beacons. Usually, it is assumed that the more fingerprints gathered, the better the radio map will represent the scenario. However, there is no clear guidance on how to carry out this process, since it depends on the scenario, the fluctuation of the signal and other parameters that must be fixed during the process (e.g., tag position, transmission power, number of transmitted beacons, *etc.*). Furthermore, we cannot take as many fingerprints as desired, since it becomes unfeasible in terms of time, especially if the indoor scenario is very large. Hence, performing the site survey is not a trivial task. Many papers, such as [25,26,27,28,29], have assumed a static tag position during the RSS measurement campaign in order to generate the radio map to characterize the spatial RSS fluctuation. Other authors have demonstrated the importance of the orientation of people with respect to the SRs, as this influences the RSS variability due to the body absorption losses, which may report an increase of up to 3–6 dB on the RSS standard deviation [30]. Furthermore, parameters, such as temperature or humidity variations produced by weather changes or the dynamics of a normal office environment (people, doors, *etc*.), can directly impact the magnitude of these irregularities on the electromagnetic channel [19]. In this paper, unlike other works, radio map fingerprints have been gathered as people stroll about the scenario (not statically), as we believe that these fingerprints will represent the electromagnetic fluctuation suffered by the signal during the online stage more realistically, when people move and the system operates in real time. Thereby, the RSS fluctuation will be better characterized by the ANN during the training process of the calibration phase.

**Figure 3:**
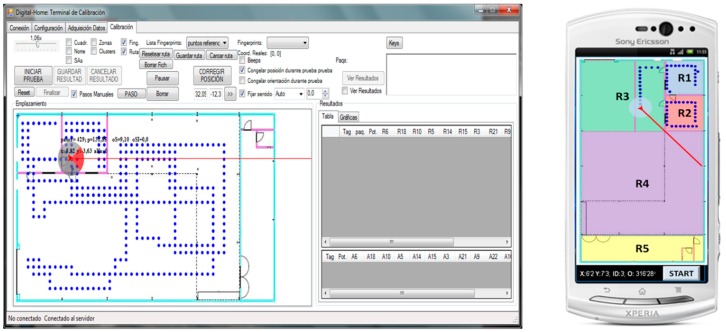
(**Left**) System management tool and (**Right**) site survey tool for Android.

Two software tools have been specifically developed to manage the site survey process efficiently in terms of time. In Figure 3, the interface of the so-called system management tool (Left) and the site survey tool (Right) are shown, which can be installed in a portable device with the Android OS (e.g., tablet or smartphone). Therefore, it is possible to control the localization server (where the system management tool is installed) remotely via WiFi from the Android device. This has allowed configuring in real time the system parameters and automatically organizing the information derived from the fingerprint generation.

**Figure 4:**
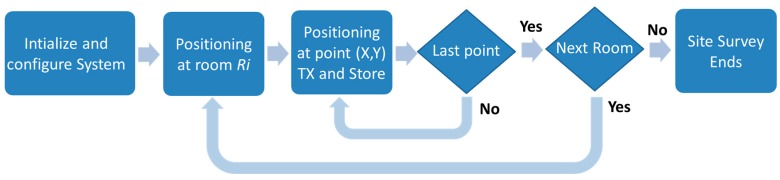
Site survey process flowchart.

In Figure 4 is summarized the process followed to generate the site survey. As the first step, the system management tool and site survey tool are started up in the server and the Android portable device, respectively. Then, the digital map of the scenario and the tag configuration (channel frequency, number of beacons, TX power, *etc.*) are loaded in the server. In the next step, a person with the Android device and the tag fixed to the body moves across each defined area/room (previously defined in the pro-project phase). Different points are chosen randomly, trying to encompass the whole area. The person moves indicating on the touch screen of the Android device the room where he/she is located. Then, a beacon burst is transmitted from the tag to generate the fingerprint associated with the current position. This process is repeated several times inside each room until no rooms remain unexplored. Finally, the server stores as text files the fingerprints in the server database with additional configuration information of interest for the investigations (RSS features, real position (room ID) of the tag, orientation, parameter values, *etc.*).

#### 4.1.2. ANN Training

After generating the radio map, the system requires a calibration phase to learn how to localize the tags from the captured RSS patterns. This is carried out by training the ANN integrated in the localization engine with the fingerprints (samples) gathered during the radio map generation.

There are different types of ANN [25,26,27,28,29]; however, here, we have considered multilayer perceptron (MLP). Although it is not demonstrated that MLP is much superior to other approaches, such as KNN or ML methods for positioning [26], one advantage of this machine is its fast response, once it has been trained in its training phase, as will be seen below. This property allows one to have a reduced system latency and, hence, process more estimations in less time. On the contrary, methods, such as KNN or ML, have to compare the input fingerprint with the whole radio map, so that computational cost exponentially rises with the size of the radio map.

**Figure 5:**
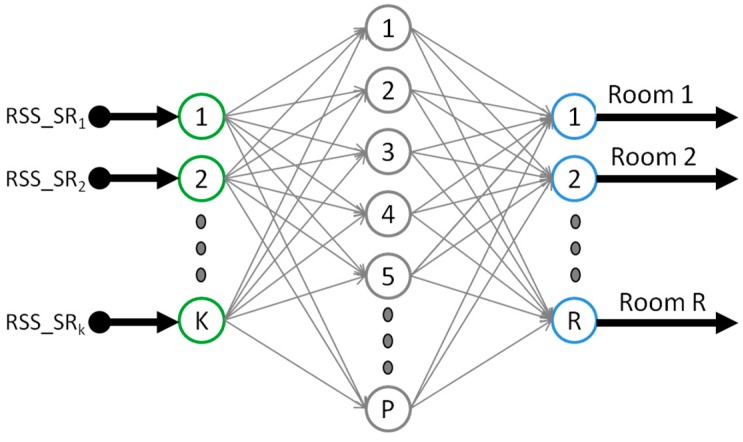
Multilayer perceptron: Proposed architecture.

The MLP is well known for being a type of universal approximator [54]. Hence, it will be possible that these ANNs learn the relation between the RSS vectors and the room ID where the tag is located (the output). The architecture of the employed MLP is sketched in Figure 5. It is composed of an input layer with *K* neurons, as many as SRs, one hidden layer with *P* neurons and an output layer with *R* neurons, as many as defined target rooms. All output neurons integrate a sigmoid transference function, which limits the output range from zero to one. To select the estimated room, the corresponding output with the highest value will be chosen.

In order to train the MLP, a training algorithm and a convergence criterion to stop the process when the goal is reached must be chosen. The training samples are the radio map fingerprints. Then, the MLP will learn to map between RSS vectors and the associated spatial locations. To do so, a supervised training algorithm will update iteratively the weights associated with each neuron, optimizing them to minimize a previously-defined cost function (usually the mean square error (MSE)). This is done until the convergence condition is met. If the radio map is sufficiently good and an appropriate architecture has been selected, the training will be correctly performed, and therefore, classification of new RSS patterns never seen before by the MLP (not presented during the training phase) should be correctly classified within the proper room. This property is called generalization, and it is what makes these mathematical tools really attractive for complex classification problems. There are dozens of training procedures based on different algorithms, such as the gradient-descent method, Levenberg–Marquardt (LM) or the classical backpropagation algorithm [54]. Furthermore, some stop training criteria, such as regularization or early-stopping, have been analyzed in order to avoid overfitting and improve generalization [54]. In this case, we have employed the Levenberg–Marquardt algorithm combined with the early-stopping method for its fast convergence and the good generalization results obtained [55]. The MLP architecture (*i.e.*, the transference function, number of hidden layers and neurons per layer) has been estimated from a trial and error test; again, as in the site survey process, no clear guidance can be found in the literature. These parameters also depend on the number of inputs/outputs defined, although it has been observed that one hidden layer with *P* = 16 neurons will be enough for the tests performed in this work, as will be seen in the experiments’ section.

Furthermore, notice that for the ANN training, the fingerprints have been pre-filtered before employing them as ANN inputs. As mentioned before, each fingerprint is described by the temporal *N* × *K* R matrix of RSS values. From this matrix, the outliers were filtered out, and missed data from out of range of the SRs was substituted by a predefined value (the sensitivity of the hardware [23,27]). Then, the real input of the MLP was the averaged RSS calculated from the *N* beacons transmitted by the tag. This pre-filtering function is the same as the one integrated in the pre-filtering layer of the localization engine.

Finally, after the training phase, the overall localization engine is tested, with the trained MLP incorporated into the classifier layer. Tests will be performed in the scenario to estimate the localization error in each defined room. If the error goal has been properly reached during the MLP training process and fits the service quality required in the pre-project phase, then the system will be considered ready to operate in the online stage, and the setup will be over. If not, the site survey will be repeated, to add more fingerprints to the radio map (e.g., repeating measurements in rooms where the localization system is mostly failing), remaking the calibration process.

### 4.2. Planning Phase

The planning phase aims to give a first approach to the number and position of the required SRs before deploying the system in the real scenario. For every fingerprinting-based ILS, it is desired to optimize these parameters to achieve better accuracy with the lowest cost possible. This is a task that is being pursued by several authors, but its analysis is not easy because of the complexity of radio propagation indoors and the number of variables to take into account (antenna radiation pattern, scenario model, antenna orientation and position, *etc.*). Only a few have dared to give some approaches based on genetic algorithms, but considering simple assumptions and propagation models [56,57].

In order to perform the planning phase, a simulation tool and a set of qualitative criteria or helping “tips” have been considered. The following planning criteria for the initial positioning of the SRs are listed below:

- Consider valid locations to place the SRs; the antennas usually will be located next to walls/roofs or places where it is possible to fix their position without hindering the normal movement of people.
- A trade-off between accuracy and cost must be taken into account; the more antennas, the greater the accuracy [15,16,17,29,30], but also, a higher cost is incurred.
- The whole scenario should be connected by at least one SR with 90% (connectivity criterion). Omnidirectional antennas are useful to report wide coverage regions, while directional antennas can be added to give coverage to more specific zones.
- In order to strengthen the accuracy in a specific room/area, directional antennas can be positioned, oriented toward that direction. Thus, a stronger RSS should be captured by that directional antenna when the tag is located in the pointed area, generating more robust RSS fingerprints.
- Antennas must be separated enough to avoid similar RSS measurements.

These criteria have been extracted empirically from the experimental tests conducted and also from results obtained in previous works [24,25,26,27,28,29,30]. Finally, note that the planning process will be iteratively repeated until the system achieves a suitable performance in the whole scenario under consideration of all of the defined rooms.

#### 4.2.1. Directional Antennas

**Figure 6:**
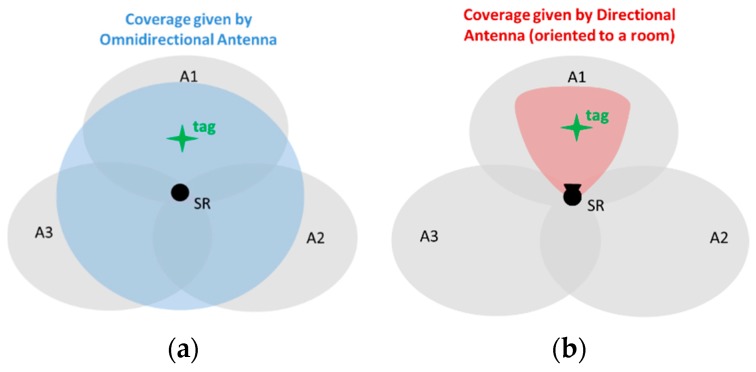
Scenario defined with three areas: A1, A2 and A3. Coverage comparison between (**a**) the omnidirectional antenna and (**b**) the directional antenna oriented toward A1.

From previous works, it has been demonstrated that the localization performance based on RSS patterns increases with the number of SRs and their strategic position [56]. Then, it is intuitive to think that the more robust the RSS fingerprints are with respect to interference noise, the easier the learning process and estimation of the tag location will be. Thereby, previous criterion *d* has been laid down on this basis. To this aim, directional antennas are proposed to help this process by virtue of their inherent spatial filtering properties. These antennas can be pointed towards a determined spatial region. Therefore, the RSS level of beacons received from non-pointed directions will be much lower than signals from the pointed ones, obtaining stronger RSS measurements from the pointed sectors where the antenna’s main beam is steered. This fact let us think about directional antennas to enhance RSS patterns associated with a particular region (area/room) of interest. This can be qualitatively understood by observing Figure 6, where a comparison between omnidirectional (Figure 6a) and directional (Figure 6b) antenna coverage inside a fictitious scenario with three defined areas (A1, A2 and A3) is shown. From this picture, it can be understood that the RSS captured by the omnidirectional antenna from a tag located in each area will be similar if compared to the RSS captured by the directional antenna mainly pointed at A1. Thus, it can be seen that the directional antenna introduces a new variable, the orientation, which allows one to increase the robustness of the RSS features in the signal space and, in theory, should enhance the robustness of the RSS patterns associated with that area, increasing the success probabilities of detecting the tag when staying at A1.

#### 4.2.2. Simulation Tool

**Figure 7:**
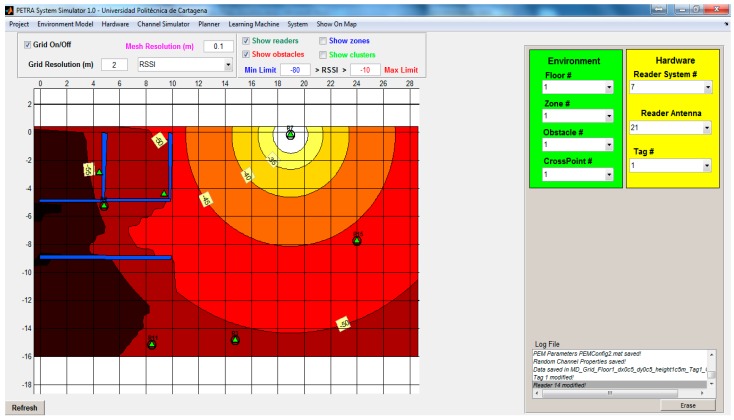
Interface of the ILS simulator.

A simulator tool was developed to aid during the planning phase. The software tool has been fully implemented in MATLAB [58] and basically can emulate the whole system, positioning the SR antennas, generating a radio map from simulated RSS fingerprints and training the ANN. Then, the performance can be approximately predicted, emulating the online stage and analyzing the performance. In Figure 7 is illustrated the interface of the simulator tool, where the main interface shows the digital map of a test scenario, and the RSS intensity is overlapped (in a range of red to white colors) for a selected SR (green triangles), visualizing the RSS distribution along the scenario.

To this end, a full model of the scenario has been developed, including walls, rooms/areas and hardware characteristics, such as the tag transmission power (*P_TX_*) or the antennas’ radiation pattern.

The RSS distribution is obtained by means of an IEEE 802.11 channel model found in [59]. This model is based on the double-slope path-loss model.

(1) $$L\left(d\right)=L_{0}+\overset{W}{\underset{w=1}{\sum }}L_{w}+10\alpha \text{log}\left(d\right)+X$$

Equation (1) includes four terms: *L_0_* (dB), which represents the signal losses at a reference distance of 1 m, *L_w_* (dB), which is the contribution due to walls (and depends on the wall material [59]), the path-loss term, which depends on *α* and *d* (the Euclidean distance between the tag and the SR, respectively) and *X*, a random variable related to the shadow fading effect, modelled by a Gaussian-type random variable with zero mean (*μ_x_* = 0) and variance *σ_x_*.

(2) $$X=\frac{1}{\sqrt{2\pi }}e^{\frac{-x^{2}}{2\sigma_{x}^{2}}}$$

Furthermore, notice that *α* and *σ_x_* will be defined as a function of the type of room (office, large office, *etc.*). The path-loss coefficient depends on distance, changing from a break point distance (*d_BP_*), as explained in [59].

Regarding the system performance evaluation for the planning phase, the figures of merit to assess the system accuracy are introduced in the next subsection.

#### 4.2.3. System Performance

Different metrics can be defined to express the ILS performance, depending on the output type provided by the system [16]. Point-based ILSs give the coordinates (*X,Y*) where the tag may be located. In these cases, the error can be calculated from the Euclidean distance between the estimated tag location (*X’,Y’*) and the exact one (*X,Y*). Furthermore, system precision is defined as the radius of the disk where the tag is positioned under a certain probability (usually established as 90%). However, in a room-level ILS case, the output of the localization engine is the room ID (*R_i_*) in which the tag is positioned. Thus, error cannot be calculated from the Euclidean distance, but as the percentage of times the true room (*i.e.*, the room where the object is located) is estimated. This is usually known as the classification success probability (*P_S_*), which can be mathematically expressed as follows:

(3) $$P_{S}=\frac{\text{Number of successful estimations}}{\text{Total estimations}}$$

In addition to *P_S_*, which represents a global system performance parameter, we will also consider the success probability associated with a pair of rooms *i, j* (*P_Sij_*), defined as the probability of selecting the room *i*, room *j* being the true room where the tag is located. Therefore, an *RxR* probability matrix (*PM*) is obtained when all combinations are calculated. This matrix is interesting because it offers detailed information about the correlation between rooms and how likely two rooms may be confused. Note that the main diagonal of the *PM* matrix (*P_Sij_* with *I* = *j*) corresponds to the success probability per room, *i.e.*, the success probability to locate the tag in room *R_i_*, when the tag is located in that room.

(4) $$PM=\left(\begin{array}{ccc} {\text{P}}_{\text{S}11} & \ldots & {\text{P}}_{\text{S}1\text{R}}\\ \vdots & \ddots & \vdots \\ {\text{P}}_{\text{SR}1} & \ldots & {\text{P}}_{\text{SRR}} \end{array}\right)$$

These metrics offer valuable information that can be employed in the experimental test phase to analyze the accuracy of the system, allowing the analysis of the system performance in a general way or, specifically, in a particular room.

#### 4.2.4. Simulation Tool

Furthermore, related to the planning phase, the connectivity concept is an important figure of merit to take into account, as it is related to the visibility of the tag from the SRs. This is an important concept for the planning phase, since the localization engine needs RSS data to infer the tag location. Hence, the more SRs are connected to the tag, the more RSSs will be captured and, hence, the more accurate will be the location estimation.

Connectivity is related to the probability of received packet (*PRP*), and statistically, it can be defined for any *k*-th SR as:

(5) PRP=nN

with *n* the number of received beacons measured by the *k*-th SR, over the total number of transmitted packets by the tag (*N*). Therefore, if an SR has not received any beacon from the tag (*PRP* = 0), that SR will be considered as “non-connected” to that location, with a *PRP* = 0.

**Figure 8:**
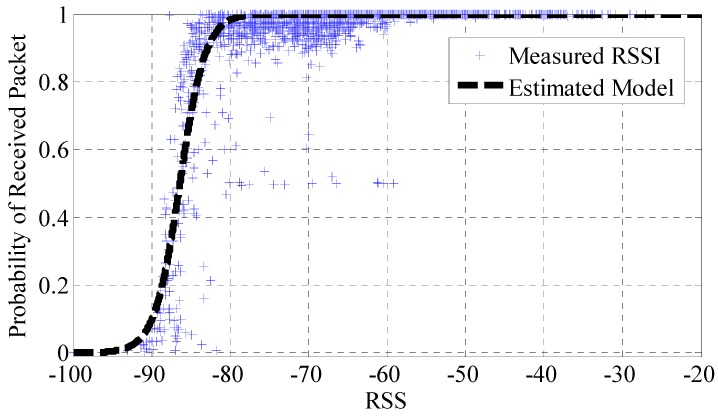
Probability of received packet (PRP) *vs*. received signal strength.

This probability is a function of the RSS, as demonstrated in Figure 8, where the *PRP* calculated experimentally is shown. This graph shows the average percentage of received beacons when an RSS level is measured. As observed, a log-sigmoid function is able to approach the cloud of measurements. It should be noted that this probability of receiving a beacon is practically *PRP* = 1 when the measured RSS is greater than −80 dBm. However, *PRP* falls down sharply till PRP = 0.5 for RSS = −86 dBm and is almost null (*PRP* = 0) when the RSS is below −90 dBm. This is in line with our expectations, as below the sensitivity of the SR hardware (which is −90 dBm), no measurements (or a very low number of them) should be obtained. Therefore, it can be derived that in order to guarantee a *PRP* > 90% in the whole scenario, the SRs must receive beacons with a minimum RSS of −83 dBm. These results reveal that the planning criteria can be laid down to meet a minimum *PRP* to ensure that useful RSS features are provided to the localization server most of the time [27].

## 5. Experimental Tests

**Figure 9:**
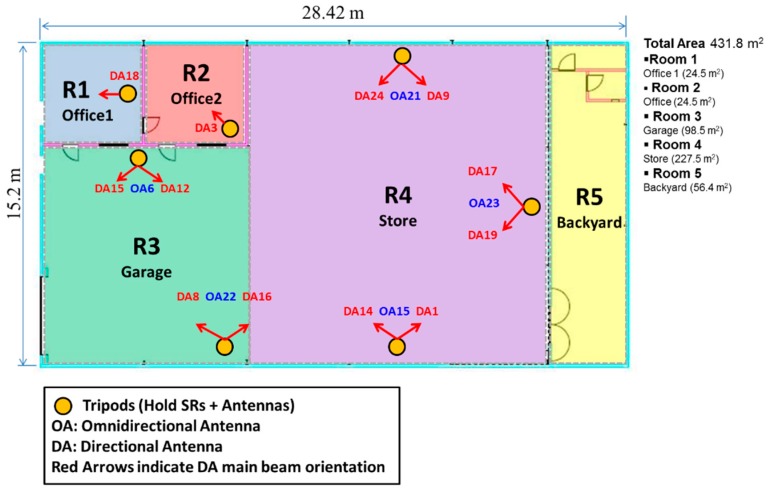
Experimental site and the sensor readers (SRs) deployed. Red arrows indicate the orientation of directional antennas.

In order to evaluate a prototype of the system, we conducted extensive experiments in an office facility with different types of areas. The location map is depicted in Figure 9 and has a size of 431.8 m^2^. This scenario represents a typical environment with furniture, people working, opening/closing doors and commercial electronic devices (computers, routers, WiFi networks, *etc.*). The map has been divided into five rooms of interest: two offices, one store, one garage and a backyard. The system has been designed with a total number of seventeen SRs positioned around the building at different reference points: five SRs with omnidirectional antennas (OA1–OA5) and twelve SRs with directional antennas (DA1–DA12). The defined rooms and the location/orientation of the SRs deployed in the scenario can be seen. Note that before the deployment of the system, the planning phase was previously performed, simulating the indoor environment and selecting the SRs’ initial positions. A connectivity of *PRP* > 90% to at least one SR from any location of the scenario was ensured.

To analyze the effect of integrating directional antennas on the system performance, different configurations of SRs with different numbers and types of antennas (directional/omnidirectional antennas) have been tested. For each configuration, the overall success probability *P_S_* and specific *PM* were calculated. Next, we describe the deployment of the prototype system on the real scenario. Later, a discussion is also given about the experimental results regarding the effect of different configurations of omnidirectional/directional antennas.

### 5.1. System Prototype

**Figure 10:**
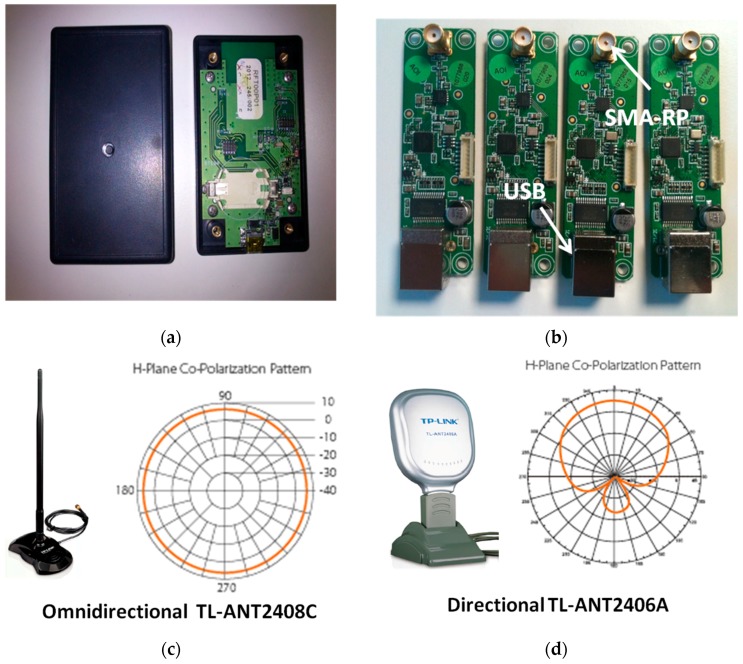
Hardware devices: (**a**) tag and (**b**) four sensor reader prototypes based on TI CC2510 modules. Employed commercial antennas (TP-Link): (**c**) omnidirectional TL-ANT2408C antenna and (**d**) directional TL-ANT2406A antenna with associated *XY*-plane radiation patterns (vertical polarization).

Figure 10 illustrates the hardware employed for the system prototype. The SR and tag prototypes (Figure 10a,b, respectively) have been designed based on the CC2510 chip [60] offered by Texas Instruments (TI). These commercial chips integrate diverse functionalities to operate as transceivers and also to capture the RSS. All related communication protocols and middleware were programmed in the C language through the CC2510DK (development kit), also offered by TI. The tag prototype incorporates an integrated PCB slot antenna with vertical polarization and a quasi-omnidirectional pattern in the *XY* plane (assuming a vertical orientation of the tag). Both devices operate in the ISM band (2.45 GHz).

**Figure 11:**
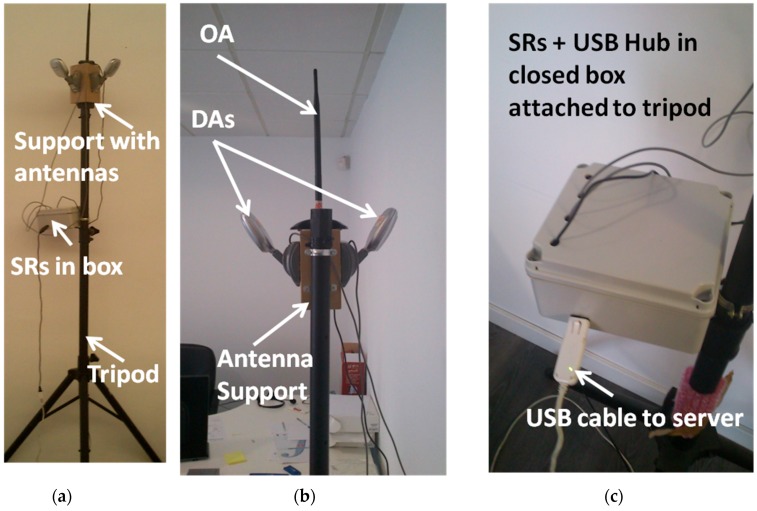
Deployed tripod examples. (**a**) Tripod with support to hold three DAs and one OA; (**b**) zoom on the support and antennas; (**c**) zoom on the box with encapsulated SRs inside; RP-SMA cables connect the SR to the antennas; the USB output cable connects the SRs to the server through a USB hub (which is embedded inside the box, with the SRs).

Each SR device has an RP-SMA (Reverse-Polarity SubMiniature version A) connector allowing it to have an external antenna plugged in. In this case, each SR can be connected to either an external omnidirectional or directional antenna. Two commercial antennas from TP-Link have been acquired for our investigations. In this case, the TL-ANT2408C omnidirectional antenna (OA) with an 8 dBi gain (Figure 10c) and the TL-ANT2406A directional antenna (DA) with a 120° horizontal half-power beam width and a 6 dBi gain (Figure 10d) were chosen. In Figure 11, the set of tripods with the SR antennas installed in order to locate the antennas in the scenario for the experiments is shown. These tripods are prepared with a support, which is able to hold up to four antennas: one omnidirectional antenna to cover 360° and three oriented directional antennas to cover sectors of approximately 120°. The tripods are two meters in height. Each tripod has a box encapsulating the SR prototypes, which are connected to the corresponding antennas via RP-SMA. In this first prototype, USB connectors have been used for the SRs to communicate with the server. Furthermore, the USB feeds the SRs hardware by its 5-V output. Future versions are being developed to employ Power over Ethernet (PoE) technology in order to get longer, less expensive and faster connection between SRs and the server. These tripods are located with the indicated antennas, according to the planning phase, at the locations depicted in Figure 9.

### 5.2. Experimental Setup

Following the deployment cycle described in Figure 2, the indoor scenario was analyzed and the radio map generated. This process was managed according to the flowchart of Figure 4 to perform the site survey process. Remember that this process is extremely relevant, since depending on how the radio map is built, it will represent in a more reliable way the irregularities and features of the indoor electromagnetic channel [17,18,26].

During the calibration phase, a person walks around the scenario, with one transmitter tag fixed to his or her chest and the Android device with the site survey tool to indicate the location and control the fingerprint generation remotely, as indicated in the previous section. The Android device used to run the site survey tool was a Sony Ericsson Xperia Neo V (Android Version 4.0.4). This device was linked to the server through a WiFi network (802.11b). It should be noted that, in order to obtain a more diverse radio map, three additional people with different heights and complexions repeated the same process. The configuration parameters used to generate the fingerprints were:

- Tag transmission power *P_TX_* = 0 dBm,
- Number of beacons per tag transmission *N* = 20,
- Refresh transmission time *T* = 500 ms.

With these settings, one fingerprint was generated approximately per second, with 20 RSS measures captured per SR. These measurements were associated with the room ID where the tag (the user) was located at that time. A total radio map with 1095 fingerprints was created for the current indoor environment approximately in 25 min, taking different random paths/pathways inside each one of the five target rooms/areas defined in the testing scenario. Furthermore, note that missed RSS data (generated by out of range SRs) were replaced by a fixed value of −90 dBm (the sensitivity of the SR receiver) for mathematical purposes, in order to keep numerical values for the ANN in the subsequent calibration phase. In Table 1 is specified the total number of samples generated per room and the sample density per square meter. From these results, note that an average density of 2.5 samples/m^2^ was kept, *R_2_* being the room with the highest density.

**Table 1:** Number of gathered fingerprints inside the scenario (431.8 m^2^).

	R_1_	R_2_	R_3_	R_4_	R_5_	Total
# Samples	86	137	264	438	170	1095
Samples/m^2^	3.5	5.6	2.7	1.9	3.0	2.5

The site survey was repeated at different time frames throughout the day (the morning, the noon and afternoon), in order to sample the indoor channel properly. This is a recommended practice, since as mentioned before, RSS measurements are quite affected by temperature and humidity variations across the day or the dynamic changes in the environment [19]. It is also worthy to note that the site survey tool was demonstrated to be very useful for the offline stage, optimizing the radio map generation, especially in terms of manpower and consuming time and without needing additional techniques to minimize radio map generation efforts, as proposed in other works [18]; a fast generation of 50 fingerprints per minute can be reached. Furthermore, for future investigations, other additional data of interest were recorded during the site survey, such as the (*X,Y*) coordinates (also provided by the user through the Android device) or the tag orientation (readily provided by the digital compass of the Android device). Furthermore, in order to speed up the process, a future version is currently being programmed, which will allow one to generate in parallel fingerprints by a collaborative method based on employing several tags at the same time to generate the radio map. This will enormously reduce the time consumption dedicated to the site survey.

After the radio map generation, the calibration phase was managed to train the ANN. For the training process, the MATLAB Neural Network Toolbox (2011 version) [58] was utilized. This library furnishes several functions to create, train and test MLPs. This process takes place in the server, which was run by an ASUS Quad-Core Intel Core i7 2.4 GHz with 4 GB RAM. Regarding the MLP architecture, a hidden layer with 16 neurons was chosen for every configuration. This parameter was obtained from trial and error, observing the minimization of the generalization error for the worst configuration, *i.e.*, when all 17 SRs are selected, which corresponds to the case with the highest number of entries to be processed by the ANN.

The calibration process must be performed for each one of the chosen SR configurations. To do so, for each selected configuration with *N’* antennas (*N’* < *N*), an ANN was trained taking the corresponding subset of *N’* RSS features from the radio map corresponding to the selected *N’* SRs employed to infer the tag location. During the ANN training process, the generated radio map was divided randomly into three sets: a training set of 766 samples (70% of the radio map) to update the network weights iteratively by the training algorithm, a test set of 220 samples (20%) to assess the generalization error of the MLP during the training process and a verification set of 110 samples (10%) in order to evaluate the stop training criterion [57,58]. Then, the ANN training process randomly initializes the numerical weights associated with each neuron. Each training step will try to find the most suitable weights to associate the input data (RSS vectors) with the desired output (room ID). For this purpose, the Levenberg–Marquardt algorithm was chosen for its memory and time efficiency [57]. Approximately, the training process reaches the convergence criterion for any SR configuration in less than 200 epochs (less than 2 min). This process was repeated several times, reinitializing MLP initial weights and the training, verification and test sets. Then, the trained network with the least generalization error (best performance) achieved was chosen. Later, the weights of the chosen trained neural network were stored in the localization engine database, to be accessed in the future during the online stage by the localization engine.

### 5.3. Test and Results

**Figure 12:**
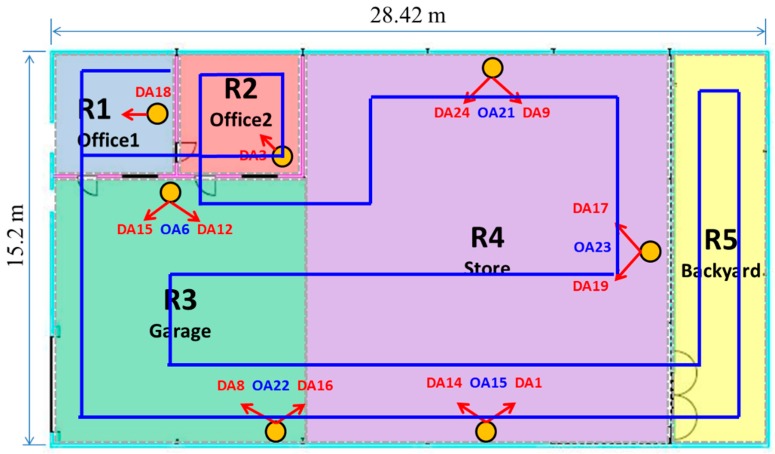
Pathway followed (in blue) by the users during experimental tests.

More than 200 SR configurations have been set for different combinations of directional and omnidirectional antennas chosen from the initial deployed set with 17 SRs. In order to evaluate the system performance for each configuration, a pathway was established to be followed by the users throughout the whole scenario, as observed in Figure 12. The pathway has been designed to go over all of the target rooms. As in the site survey process, different people carried the tag fixed to their chest to perform the tests with the tag transmitting beacons periodically as the person moves all over the defined areas. Therefore, the reliability of the system is tested in quite hard realistic conditions, taking into account that people movement and body orientation are increasing the RSS temporal fluctuation even more [30].

The pathways were followed in both directions, and the tests were repeated daily at different time frames during one week. During the tests, the system was operating in its online stage, and the tag was configured to transmit *N* = 10 beacons periodically with a shorter refresh time of *T* = 250 ms (higher *N* and lower *T* values would overload the system). The tag power transmission was *P_TX_* = 0 dBm (as previously established during the site-survey process, to ensure connectivity between the tag and all of the SRs along the whole scenario most of the time). Hence, about 500 positions were inferred for each pathway (four per second during 2 min of walking). Finally, a total of 27 tests were performed.

The probability matrix *PM* presented in Equation (4) was calculated from the test results for every SR configuration. The *P_Sij_* per each room was easily obtained from the main diagonal of the *PM*. The overall *P_S_* was also obtained applying Equation (3), dividing the total number of successful estimations over the total number of estimations carried out during the test.

Table 2 presents the results for the most outstanding SR configurations in terms of location success probability (*P_S_*). The table represents the minimum performance (per room and global) obtained for 90% of the 27 tests performed per each configuration. Furthermore, in order to give a deeper comprehension of the results presented in Table 2, Figure 13 shows a box-and-whisker plot with further information about the overall *P_S_* resulting from the 27 tests performed for each SR configuration analyzed in Table 2. This analysis has been conducted using MATLAB function “boxplot”, given the calculated *P_S_* resulting from each test. In each plot, the central mark is the median *P_S_* obtained for each configuration, the edges of the box are the 25th and 75th percentiles; the whiskers extend to the most extreme resulting *P_S_* values (not considered as outliers); and the considered outliers are plotted individually as red crosses.

**Table 2:** Success probability per room *P_Sr_* (*r*-th room) and overall *P_S_*.

#	Antennas ID	*Ps_1_* (%)	*Ps_2_* (%)	*Ps_3_* (%)	*Ps_4_* (%)	*Ps_5_* (%)	*Ps* (%)
**1**	3 OA = {2,3,4}	17	7.8	18.3	68.5	39.8	43.9
**2**	3 OA = {1,2,3}	18.2	6.7	52.3	62.1	44.6	52.5
**3**	5 OA = {1–5}	21.3	7.1	51.3	68.1	44.7	53.8
**4**	2 DA = {1,10}	67.4	36.1	36.0	65.1	47.9	54.5
**5**	3 DA = {1,5,11}	69.7	39.9	57.1	60.6	52.6	65.4
**6**	5 DA = {1,2,5,8,10}	73.5	81.1	71.4	70.4	55.1	75.7
**7**	8 DA = {3,4,5,6,7,9,11,12}	13.9	32.4	74.4	81.8	49.8	63.4
**8**	12 DA = {1–12}	72.3	85.0	77.6	82.2	62.1	80.7
**9**	3 OA = {2,3,4} + 1 DA = {1}	71.4	38.5	41.3	68.5	48.4	63.9
**10**	3 OA = {2,3,4} + 1 DA = {2}	20.6	74.0	45.1	64.3	52.2	59.9
**11**	3 OA = {2,3,4} + 2 DA = {1,2}	70.8	74.4	68.9	83.2	53.2	79.4
**12**	5 OA = {1-5} + 12 DA = {1–12}	79.4	84.0	74.6	82.5	57.1	81.0

**Figure 13:**
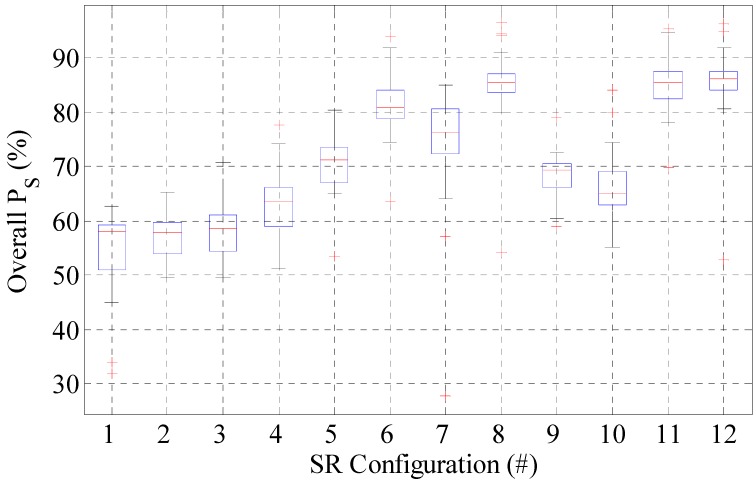
Box-and-whisker plot for the *P_S_* resulting from 27 test pathways for the configurations presented in Table 2.

### 5.4. Discussion

Comparing the overall performance obtained for every configuration, it is clearly observed that the planning of the SRs is relevant, since different configurations of SRs may result in very different performances of the system for the same scenario. It is revealed that the more SRs, in general, the better the system performance achieved, according to the conclusions of [16,29,30]. However, it has been observed that this tendency is not always accomplished. Observing several configurations with the same or even a greater number of antennas, independently of the antenna type, it is shown that the same configurations with different SRs can obtain quite dissimilar performances, even degrading it if no proper antennas are included for the tag estimation. Regarding this phenomena, in Table 1, some of the configurations can be compared, and it can be conclude that adding more antennas is not always a feasible solution to enhance *P_S_*. This is clearly appreciated comparing Configuration #1 *vs*. #2, with three OAs each, for which different performances (44% and 52%, respectively) with the same number of OAs were obtained. Furthermore, this can be observed if we compare Configuration #2 vs. #3, where the performance keeps constant with 53%, despite that two more OAs being employed for the location estimation in Configuration #3. Furthermore, observing Configuration #6 against #7, with five and eight Das, respectively, the performance decreases from 75.7% to 63.4%. From these observations, it can be derived that the increase of the number of antennas does not always enhance the performance, but planning must be also taken into account properly, since adding antennas arbitrarily might even produce the opposite effect.

Regarding the inclusion of directional antennas, the results also confirm our assumptions about the spatial filtering properties to enhance the fingerprints’ robustness and quality. It can be observed how the integration of directional antennas may improve performance in some rooms. This is proven by observing the performances obtained for Rooms 1 and 2 (corresponding to two offices with a similar area of 24.5 m^2^) in Configurations #1, #9, #10 and #11. The employment of just OAs in Configuration #1 does not improve the performance inside those rooms, it being not possible to distinguish between them, and attaining a poorer performance inside these areas: *P_S1_* = 17% and *P_S2_* = 7.8%. However, the insertion of a directional antenna may improve performance, as can be noted comparing Configurations #9 and #10, where DA1 and DA2 are included respectively, increasing significantly the performance in R1 and R2 up to *P_S1_* = 71.4% and *P_S2_* = 74%, respectively, with respect to the firs cited configuration with only three OAs. Finally, DA1 and DA2 are combined with three OAs in Configuration #11, and a significant improvement for rooms R1 and R2 is achieved (*P_S1_* = 70.8% and *P_S2_* = 74.4%) at the same time, increasing the overall performance up to 79.4%. This example clearly demonstrates how the insertion of Das, suitably oriented and positioned, may further improve the performance in certain rooms due to the spatial discrimination enhancement associated with directive antennas, confirming our initial hypothesis.

It should be also noted that comparing previous Configuration #11 (with three OAs and two DAs) with #6 (with 12 DAs) and #12 (with five AOs and 12 DAs), a similar performance is obtained, corroborating that the inclusion of more antennas is not worthwhile in this experimental case. Hence, it is clearly demonstrated how a properly deployed five-antenna configuration (#11), which combines three OAs and two DAs, is able to obtain almost the same performance as for configurations with 12 (#6) and 17 (#12) antennas, respectively. These observations lead us again to remark on the importance of the planning phase and how properly positioning and orienting the SR antennas, the number of required SRs can be drastically reduced to optimize the performance of the system with the minimum number of antennas.

Finally, notice that the 25th and 75th percentiles (limits of the blue boxes in Figure 13) are really close for Configurations #6, #11 and #12. This means that the performance achieved for these configurations is stable between those values (about 82.5% and 87.5% for Configuration #11) for almost every test performed.

To sum up, several conclusions can be highlighted from this study:

- It has been observed that the monotonically-increasing tendency of accuracy (successes probability) with the number of antennas is not totally true. The planning phase (antennas’ position) affects the performance of the system severely.
- DAs may improve the accuracy of the system if they are properly deployed and oriented, allowing a better distinction between target areas (rooms) without excessively increasing the number of antennas. During the experiments, this is demonstrated with a configuration with five antennas (three AOs and two ADs), which results in a similar performance as the one obtained by 17 antennas (five AOs and 12 ADs).
- The above point endorses that the integration of DAs increases the fingerprint quality, making RSS patterns more robust against noise and more easily recognizable by the MLP algorithm.

From these remarks, it is evidenced that the addition of directional antennas enhances the performance of RSS-based systems if SRs are deployed properly. However, as mentioned previously, RSS-based systems are linked to an inherent limitation in terms of maximum accuracy due to the complex unpredictable variability of the RSS [16], that together with the ANN error, makes the ANN not be able to predict the room location with 100% success.

At this point, it would be also interesting to compare our results with the state-of-the-art woks. To do so, we have taken into account the most recent references found in the literature related to room-level RSS-based positioning [21,22,23]. If we try to compare our best results in terms of accuracy and number of antennas directly, which has been for Configuration #11 *Ps* (90%) = 79.4% (with five antennas), it seems we have *a priori* inferior results compared to the over 90% average success results obtained in [21,22,23]. However, it is important be take into account several points to compare our results to others:

- The metric we have employed is quite demanding, as we calculate the minimum *Ps* obtained for 90% of the tests, which gives an estimation of the system reliability for 90% of the time.
- The work in [21,22,23] provided average *Ps*, which is the mean of all of the tests conducted. This metric is much poorer, as it does not talk about the reliability of the system and takes into account outliers. In fact, if we averaged the *Ps* results for the 27 tests done with Configuration 11, our average success *Ps* (average) = 86% (see the red line in the middle of the box for Configuration #11 in Figure 13), which seems to be much better, but it has no information about reliability.
- However, another factor to take into account is the test conditions. The covered area, as the number of SR in [21,22,23], is quite different. For example, in [23], 17 omnidirectional antennas were employed.
- Furthermore, no information regarding the conditions of the tag or the scenario is given (e.g., some questions related to the static, fixed orientation of the tag during transmission/reception; people presence in the environment).

In this paper, we have employed a simple MLP-based indoor positioning system to analyze the directional antennas’ effect on the positioning performance. However, it is worthy to note that to overcome the system limitations, extra information from other data sources (e.g., accelerometers or other sensors, such as infrared) or building geometry, could be integrated in the post-processing layer engine [32], in order to refine the raw estimation obtained from the classifier layer, although at the expense of increasing system complexity and computational cost. To this aim, several algorithms and techniques have already been largely studied [21,22,23,31,32,33,34,35] that could be combined with our system for future studies.

To finalize, another point that is interesting to remark on before concluding this section is that similar conclusions are expected if another classifier, such as KNN, ML or SVM, is employed in the location engine, instead of the MLP. The authors obtained similar results to the ones presented in Table 2 and Figure 13 employing the KNN classifier; however, for the sake of space, and to focus on the principal concern of this paper, we have presented the results for an MLP, which is a well-known classifier that has been demonstrated to be stable and advantageous compared to others in terms of time response (which is quite important to run the prototype during the tests).

## 6. Conclusions

The present paper addresses the integration of directional antennas to enhance the performance of receive signal strength (RSS) fingerprinting-based indoor localization systems (ILS). A room-level RSS fingerprinting-based ILS has been developed for research purposes, with a localization engine based on an artificial neural network (ANN) to infer the tag position. The system architecture is based on a sensor network composed of a set of *K* sensor readers (SRs), each one connected to an external omnidirectional or directional antenna, which are in charge of measuring the RSS from the beacons transmitted by the tag. A centralized server will infer the tag position in real time from the RSSs gathered by the SRs. A full deployment process of the sensor network is described. A planning phase was previously performed, aided by our own simulation tool, to estimate the number and position/orientation of omnidirectional/directional antennas in order to optimize the system performance. After planning, the physical deployment of the sensor network is chosen, and a site survey process is conducted to generate a radio map with fingerprints that rigorously characterizes the indoor environment. A final calibration phase is used to train the ANNs with the radio map data in order to infer the possible tag positions from the RSS fingerprints. A prototype has been deployed in a real scenario of 432 m^2^ used as the experimental testbed. The scenario represents the conditions of typical office environment activity. Different combinations of omnidirectional and directional antennas have been intensively studied to analyze the effect of antenna type to increase the probability of positioning the tag in the true room. A radio map with more than 1000 fingerprints was generated in an efficient way, by the aid of our own software tools developed in C#, Java and MATLAB, allowing generating fingerprints very quickly and cost-effectively. Furthermore, different test pathways performed by different people were used to better characterize and evaluate the system performance during the experiments.

In general, the results demonstrate our initial hypothesis, which established that adding directional antennas, suitably oriented and positioned, introduces a new degree of freedom (and novelty), which increases the quality of the RSS fingerprints, ultimately enhancing the room discrimination realized by the ANNs. This way, it has been demonstrated that in most of the configurations evaluated, the localization performance could be improved if appropriate sets of directional and omnidirectional antennas were chosen. A summary of the most successful results allows us to conclude that the performance can be improved in certain rooms using properly-positioned/oriented directional antennas, without affecting the performance in other spatial regions and, therefore, improving the overall system performance. Furthermore, results also demonstrate that using properly-planned configurations combining omnidirectional and directional antennas would allow maintaining good performance while the number of needed antennas is dramatically reduced, therefore saving on economic costs.

Future works will be focused on the introduction of new low-cost planar and multi-beam directive antennas. These antennas will be able to sectorize space with three or six narrow beams (low beam width) in order to get better results, without economically jeopardizing the solution and gaining the feasibility of the RSS fingerprinting-based ILSs.
